# Optimisation of COVID‐19 diagnostic pathways in acute hospital admissions to prevent nosocomial transmission

**DOI:** 10.1111/crj.13530

**Published:** 2022-08-03

**Authors:** Robert Livingstone, Alexander Woodhead, Megha Bhandari, James Dias, Trevor Smith, Tom Havelock, Matthew Stammers

**Affiliations:** ^1^ University Hospital Southampton NHS Foundation Trust Southampton UK; ^2^ CIRU – Clinical Informatics Research Unit Blood and Transplant Unit Southampton UK

**Keywords:** chest X‐ray, COVID‐19 testing, cross infection, reverse transcription polymerase chain reaction

## Abstract

**Introduction:**

In the management of acute hospital admissions during the COVID‐19 pandemic, safe patient cohorting depends on robust admission diagnostic strategies. It is essential that screening strategies are sensitive and rapid, to prevent nosocomial transmission of COVID‐19 and maintain patient flow.

**Methods:**

We retrospectively identified all COVID‐19 positive and suspected cases at our institution screened by reverse transcription polymerase chain reaction (RT‐PCR) between 4 April and 28 June 2020. Using RT‐PCR positivity within 7 days as our reference standard, we assessed sensitivity and net‐benefit of three admission screening strategies: single admission RT‐PCR, composite admission RT‐PCR and CXR and repeat RT‐PCR with 48 h.

**Results:**

RT‐PCR single‐test sensitivity was 91.5% (87.8%–94.4%) versus 97.7% (95.4%–99.1%) (*p* = 0.025) for RT‐PCR/CXR composite testing and 95.1% (92.1%–97.2%) (*p* = 0.03) for repeated RT‐PCR. Net‐benefit was 0.83 for single RT‐PCR versus 0.89 for RT‐PCR/CXR and 0.87 for repeated RT‐PCR at 0.02% threshold probability.

**Conclusion:**

The RT‐PCR/CXR composite testing strategy was highly sensitive when screening patients at the point of hospital admission. Real‐world sensitivity of this approach was comparable to repeat RT‐PCR testing within 48 h; however, faster facilitating improved patient flow.

## Graphical abstract

RT‐PCR/CXR composite testing was highly sensitive when screening patients at the point of hospital admission for COVID‐19 infection. Real‐world sensitivity of this approach was comparable to repeat RT‐PCR testing within 48 h; however, faster facilitating improved patient flow.

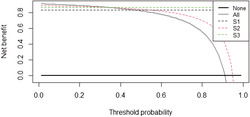

## 1 INTRODUCTION

Coronavirus disease 2019 (COVID‐19) has introduced new challenges in safely cohorting hospitalised patients. Accurate diagnostic methods and robust protocols are essential to prevent nosocomial transmission.

Hospital‐acquired COVID‐19 mortality was cited as 36%.^1^ Diagnostic uncertainty can lead to isolation facility tie‐up, significant loss of bed capacity and ‘flow’.

The gold standard for COVID‐19 diagnostic screening has to date been reverse transcription polymerase chain reaction (RT‐PCR).^2^, ^3^ In meta‐analyses, pooled sensitivity of the gold‐standard RT‐PCR has been cited as 86% and 89%,^4^, ^5^ while sensitivity of chest computed tomography (CT) has been shown to be higher at >90%.^3^, ^5^ CT carries comparatively high radiation dosage and is resource‐intensive; as such, it is a poor screening substitute in most settings. The sensitivity of chest X‐ray (CXR) has been variably reported, with some studies suggesting near‐comparability with CT.^6^

We performed a retrospective comparison of three COVID‐19 admission screening strategies in real‐world clinical practice: single admission RT‐PCR, composite admission RT‐PCR and CXR and repeat RT‐PCR with 48 h.

## 2 MATERIALS AND METHODS

### 2.1 Study setting

This study was undertaken at UHS, a tertiary centre within the United Kingdom.

### 2.2 Cohort selection

All COVID‐19 suspected and positive cases between 4 April and 28 June 2020 were identified. We excluded those receiving RT‐PCR testing prior to 4 April 2020, when the previously used Public Health England RNA‐Dependent RNA Polymerase (PHE RdRp) single gene target screening assay was discontinued following recognition of its lower sensitivity.^7^ Replacement targets included the World Health Organisation (WHO) E gene target^8^ and the Centers for Disease Control and Prevention (CDC) N2 target.^9^

We excluded all suspected hospital‐acquired cases as defined by NHS England^10^ and those without a CXR performed within 24 h of admission.

**FIGURE 1:**
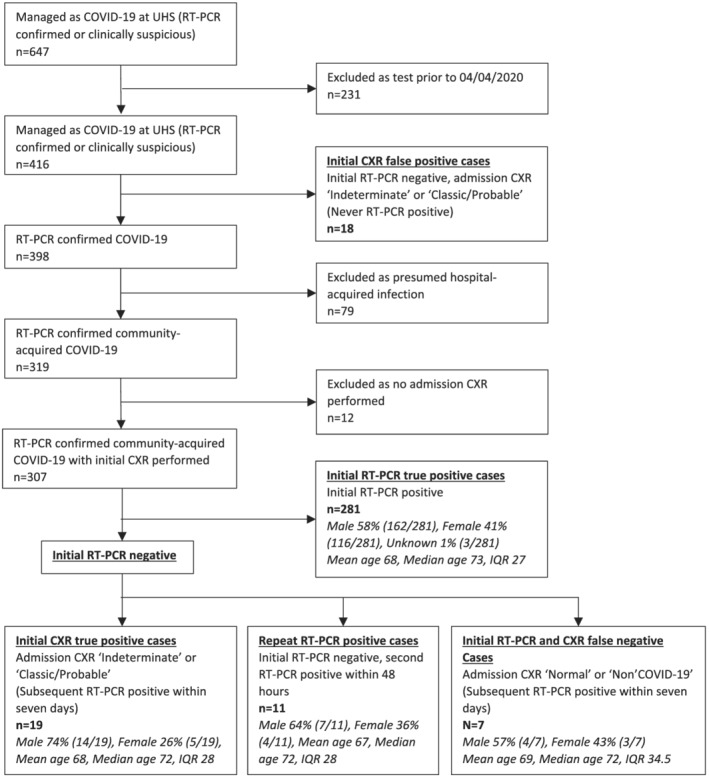
Cohort selection flowchart

The resultant cohort of community‐acquired COVID‐19 cases was split by result of initial RT‐PCR and initial CXR. CXR result was defined by radiologist report as per national British Society of Thoracic Imaging (BSTI) reporting guidelines.^11^ We identified the following key subgroups (see Figure 1 for flowchart):

[*Initial RT‐PCR true positive cases*]: Initial RT‐PCR positive.

[*Initial CXR True Positive cases*]: Initial RT‐PCR negative but admission CXR ‘Indeterminate’ or ‘Classic/Probable’ for COVID‐19 infection as defined by national BSTI CXR reporting guidelines). Subsequently testing RT‐PCR positive for COVID‐19 within 7 days.

[*48 hour* repeat *RT‐PCR positive cases]*: Initial RT‐PCR negative, second RT‐PCR positive within 48 h.

[*Initial RT‐PCR and CXR false‐negative cases*]: Initial RT‐PCR negative with admission CXR ‘Normal’ or ‘Non‐COVID‐19’ as defined by national BSTI CXR reporting guidelines. Subsequently testing RT‐PCR positive within 7 days.

[*Initial CXR false‐positive cases*]: Initial RT‐PCR negative, admission CXR ‘Indeterminate’ or ‘Classic/Probable’ for COVID‐19 infection as defined by national BSTI CXR reporting guidelines. Never testing RT‐PCR positive.

### 2.3 Data collection, handling and processing

Clinical and demographic data for all cases were retrospectively acquired. All RT‐PCR and plain‐film radiological investigations were extracted. All data was appropriately handled during analysis.

### 2.4 Statistical analysis

We assessed the real‐world sensitivity and net‐benefit of three COVID‐19 admission screening strategies:

**Strategy 1:** Single admission RT‐PCR.

**Strategy 2:** Combined admission RT‐PCR and CXR.

**Strategy 3:** Repeat RT‐PCR testing within 48 h.

Kruskal–Wallis, McNemar's exact test with Bonferroni correction and logistic regression were used to model the three strategies using R (4.0.3) and Python 3.8+ packages. All *p* values were reported at the 0.05 threshold.

Discrimination, calibration and clinical model utility were assessed using decision curve analysis.

In simple terms, net‐benefit is an increasingly reported decision analytic measure that puts benefits and harms on the same scale and more closely reflects the realities of clinical practice as expounded in this BMJ article.^12^ Net‐benefit is calculated using the below formula:

$$\text{Netbenefit}=\frac{\text{Truepositives}}{N}-\frac{\text{Falsepositives}}{N}\times \frac{p_{t}}{1-p_{t}}$$

## 3 RESULTS

Three hundred seven patients with positive RT‐PCR within 7 days of admission (community‐acquired COVID‐19 cases) were screened with both admission RT‐PCR and CXR. Fifty‐nine percent were male, average age: 68. There was no significant difference between groups (*p* = 0.199). See Figure 1 for a detailed breakdown of the cohort selection process and cohort characteristics.

### 3.1 Strategy 1

The sensitivity of initial RT‐PCR was 91.5% (*n* = 281/307, 95% CI 87.8%–94.4%).

### 3.2 Strategy 2

Composite clinical sensitivity of admission RT‐PCR and CXR was 97.7% (*n* = 300/307, 95.4%–99.1%), which was significantly better than RT‐PCR alone at the *p* < 0.05 threshold (*p* = 0.025).

### 3.3 Strategy 3

Sensitivity of repeat RT‐PCR testing within 48 h was 95.1% (*n* = 292/307, 92.1%–97.2%), which was also significantly better than RT‐PCR alone at the *p* < 0.05 threshold (*p* = 0.03).

**FIGURE 2:**
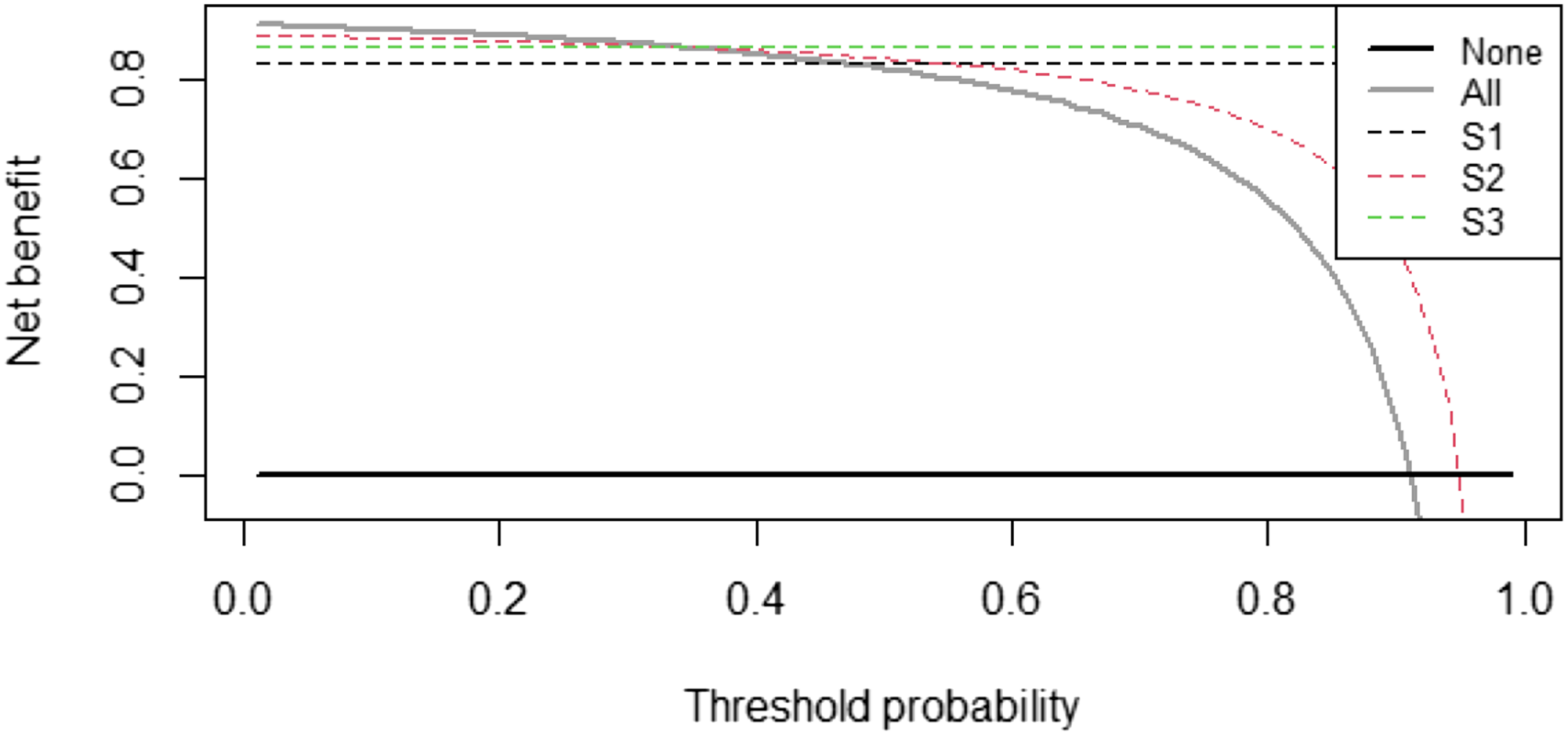
Net‐benefits model for single admission RT‐PCR (strategy 1, ‘S1’) versus combined admission RT‐PCR and CXR (strategy 2, ‘S2’) versus repeat RT‐PCR testing within 48 h (strategy 3, ‘S3’)

Strategy 1 performed comparatively poorly (Figure 2) with a net benefit of only 0.83 at the 0.02 threshold (very low clinical tolerance for diagnostic uncertainty). Strategies 2 and 3 were more similar at this threshold, with net benefit of 0.89 and 0.87, respectively. However, time‐to‐decision was reduced by over 24 h by strategy 2 versus strategy 3. Mean time to second RT‐PCR was 26.5 h in strategy 3, while in strategy 2, the CXR always occurred prior to receipt of the first RT‐PCR lab result.

## 4 DISCUSSION

We report a real‐world sensitivity of initial RT‐PCR for detecting COVID‐19 in acute hospital admissions of 91.5%. This is comparable with the results of two pooled sensitivity studies,^4^, ^5^ which report sensitivities of 86% and 89%, respectively, for RT‐PCR. Composite sensitivity of initial RT‐PCR and initial CXR was 97.7%, superior to RT‐PCR alone, indicating that single RT‐PCR alone missed 6.2% of cases. The RT‐PCR/CXR composite test strategy was thus effective at screening patients at the front‐door, dramatically reducing the risk of nosocomial infection. Real‐world sensitivity of this approach was comparable to repeat testing within 48 h, however much quicker allowing safe patient cohorting and efficient patient flow. The sensitivity of this composite approach was near comparable with published data for CT,^3^, ^5^ however notably without the radiation dosage, resource burden or cost associated with this modality. This approach therefore would be feasible in resource‐poor environments where CT is unavailable and repeat RT‐PCR costly.

We identified a small number of subsequently positive patients who would not have been detected with any screening methodology, presenting a significant risk of nosocomial transmission. Within this cohort, we noted two patients with underlying cognitive impairment, one immunosuppressed, one neurological presentation^13^ and one gastrointestinal. Clinical judgement must therefore remain an essential complement.

Limitations include this being a single centre study and not a clinical trial. Also, not all suspected cases of COVID‐19 who were initially RT‐PCR negative received repeat testing within 48 h. This could have resulted in an underestimation of the true sensitivity of strategy 3.

We analysed a real admissions process, as opposed to individual diagnostic tests in a simulated or laboratory environment. Reliable RT‐PCR testing was used, including highly sensitive rapid point‐of‐care testing still not reliably available in many Western healthcare settings.^7^

We have managed to demonstrate excellent performance of combination testing with initial RT‐PCR and CXR in action and have produced results directly clinically applicable, particularly within resource poor contexts even today.

## 5 CONCLUSIONS

It remains vitally important that we are able to appropriately cohort patients while preventing nosocomial transmission of COVID‐19. We have demonstrated that combination admission RT‐PCR/CXR is superior to single RT‐PCR alone at reducing risks of nosocomial COVID‐19 transmission.

## CONFLICT OF INTEREST

None from any of the authors.

## ETHICS STATEMENT

This work was performed as part of a service evaluation approved by the University Hospital Southampton (UHS) Caldicott Guardian and COVID‐19 Clinical Lead.

## AUTHOR CONTRIBUTIONS

RL is the principal author. AW and MS are responsible for the analysis and statistics. MB and JD are contributing authors. MS and TH are the lead clinicians and originators of the study. TS is the contributing and reviewing author.

## Data Availability

Raw data were generated at University Hospital Southampton. Derived data supporting the findings of this study are available from the corresponding author (RL) on request.
